# Does ophthalmic-grade silicone oil possess antimicrobial properties?

**DOI:** 10.1186/s12348-019-0187-6

**Published:** 2019-11-01

**Authors:** Vivek Pravin Dave, Joveeta Joseph, Priyanka Jayabhasker, Rajeev Reddy Pappuru, Avinash Pathengay, Taraprasad Das

**Affiliations:** 1 Smt. Kanuri Santhamma Center for Vitreoretinal Diseases, LV Prasad Eye Institute, Hyderabad, Telangana India; 2 Jhaveri Microbiology Center, LV Prasad Eye Institute, Hyderabad, Telangana India; 3 Retina and Uvea Service, GMRV Campus, LV Prasad Eye Institute, Visakhapatnam, India

**Keywords:** Silicone oil, Antimicrobial activity, Endophthalmitis

## Abstract

**Purpose:**

To test the antimicrobial properties of silicon oil (Aurosil 1000 cSt, Aurosil Plus 5000 cSt) on in vitro growth of common microorganisms causing endophthalmitis.

**Materials and methods:**

*Staphylococcus aureus*, *Staphylococcus epidermidis*, *Pseudomonas aeruginosa*, multi-drug resistant (MDR) strain of *Klebsiella pneumoniae*, *Escherichia coli*, *Candida albicans*, and *Aspergillus flavus* were prepared to 0.5 McFarland turbidity. The bacteria and fungi were inoculated into the silicone oils, brain heart infusion (BHI) broth for bacteria and Sabouraud dextrose agar (SDA) broth for fungi, respectively, and cultured aerobically for 30 days. From each sample, 10 μl was plated onto nutrient agar and potato dextrose agar (PDA) for testing growth of bacteria and fungi respectively. Cultures from specimens, overnight incubation, and CFU counting were repeated on days 1, 3, 5, 7, 14, 21, 24, and 30. Negative controls were brain heart infusion and physiologic saline as well as silicone oils without any inoculations.

**Results:**

All bacteria showed a decrease in CFUs by the fifth day and eliminated between 21 and 30 days in silicone oil. The silicon oil, irrespective of its viscosity, had only fungistatic effect up to 30 days. Colony-forming units of microorganisms remained stable in physiologic saline during the study. In BHI and Sabouraud broth, both bacteria and fungi showed a growth pattern that was compatible with the growth curve of microorganisms.

**Conclusion:**

Medical-grade silicone oil used in ophthalmology exhibited in vitro bactericidal and fungistatic activity over 30 days. Insertion of silicone oil in vitrectomy for endophthalmitis, when required, could supplement the antimicrobial activities of intravitreal antibiotics in management of endophthalmitis.

## Background

Silicone oils (SO) constitute a group of inert, clear, and hydrophobic polymers, chemically derived from siloxane [1, 2]. Chemically, they are composed of organic and inorganic polymers having repetitive siloxane (Si–O) units. The most common usage of SO is in complex retinal detachment surgery such as proliferative vitreoretinopathy (PVR) and giant retinal tears. Silicone oil is also used in extreme cases of endophthalmitis. Few recent studies have alluded to the antimicrobial properties of silicone oil [3–6]. The conclusions from those studies have been equivocal with evidence both in favor and against the role of silicone oil having antimicrobial activity.

We designed an in vitro study to evaluate the antimicrobial properties of two viscosities of medical-grade silicone oil used in ophthalmology against the common microorganisms causing endophthalmitis [7–9].

## Methods

This was a prospective in vitro study conducted at the ocular microbiology laboratory of the institute at Hyderabad, India. Appropriate Institutional Review Board approval was taken. We chose two viscosities of silicone oil, 1000 cSt and 5000 cSt from one manufacturer (Aurolab, Madurai, India). The bacterial laboratory isolates included common bacteria—*Staphylococcus aureus*, *Staphylococcus epidermidis*, *Escherichia coli*, *Klebsiella pneumoniae*, *Pseudomonas aeruginosa*—and common fungi—*Candida albicans and Aspergillus* flavus. The silicone oils (Aurosil 1000 cSt and 5000 cSt) were inoculated with both bacteria (*Staphylococcus aureus*, methicillin-resistant *Staphylococcus aureus (*MRSA), *Pseudomonas aeruginosa*, multi-drug resistant (MDR) *Klebsiella pneumonia*) and fungi (*Candida albicans* and *Aspergillus flavus*). The microorganisms were suspended in physiologic saline to get 0.5 McFarland turbidity (1 × 10^8^ cfu/ml). Two samples of 0.1 ml were inoculated into both the 0.9-ml silicone oils and 0.9-ml brain heart infusion broth for bacteria and Sabouraud dextrose agar (SDA) broth for fungus to serve as positive control. Negative controls were brain-heart infusion and physiologic saline as well as silicon oils without any inoculations. In both tested oils and control media, the microbes were cultured aerobically for 21 days, bacteria at 37 °C, and fungi at 30 °C. The antimicrobial effect of both silicone oils was determined by the growth capability of the microorganism. Before sampling, the tubes were carefully vortexed at 2500 rpm until bacteria/fungus and silicone oil mixture with evenly distributed microorganisms was obtained. Samples were taken from these liquids at given intervals (1, 3, 5, 7, 14, 21, 24, and 30 days) and inoculated and spread on Müeller-Hinton agar or SDA. After 24 h of aerobic incubation at 37 °C (bacteria) and 48 h at 30 °C (yeasts and fungi), the grown colonies were be counted and the numbers of colony-forming units in 1 ml (CFU/ml) determined.

## Results

Both the silicone oils demonstrated a significant decrease in bacterial load at day 5 against all bacteria tested, and the antibacterial effects continued for 21 days before declining to zero levels at the end of 1 month. Similar pattern was not seen with fungi in silicone oil. The fungi remained static, neither increasing nor decreasing.

**Fig. 1:**
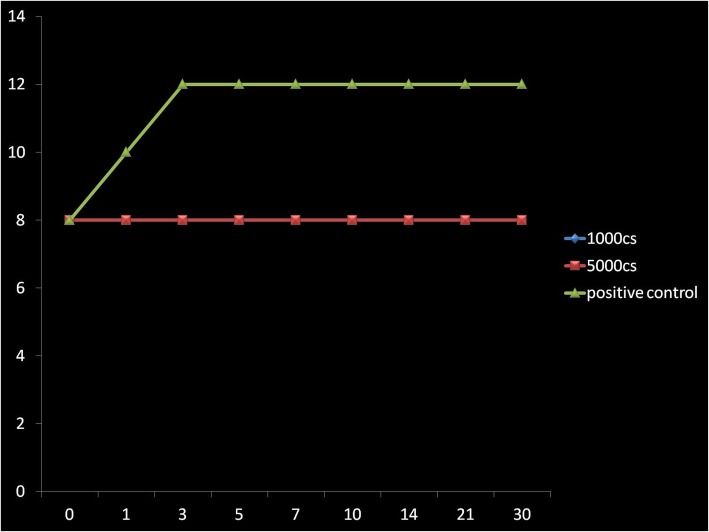
Graph showing the growth pattern of *Aspergillus*. *X*-axis is in days, *Y*-axis has CFU (1 × 10^10^)

**Fig. 2:**
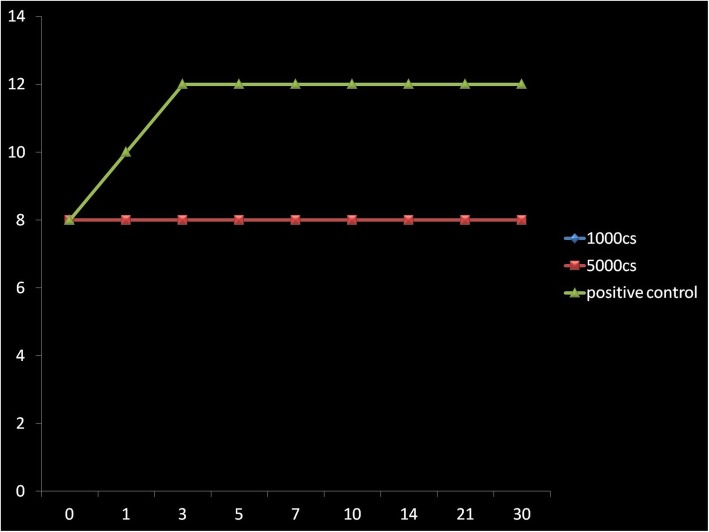
Graph showing the growth pattern of *Candida albicans*. *X*-axis is in days, *Y*-axis has CFU (1 × 10^10^)

**Fig. 3:**
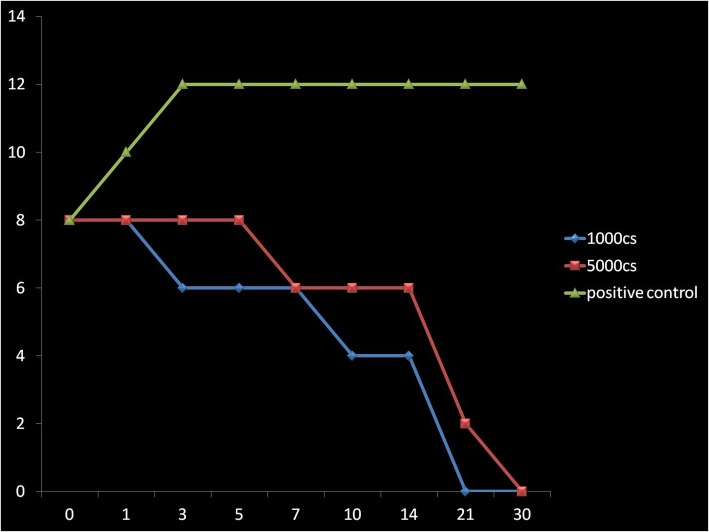
Graph showing the growth pattern of *Klebsiella pneumoniae*. *X*-axis is in days, *Y*-axis has CFU (1 × 10^10^)

**Fig. 4:**
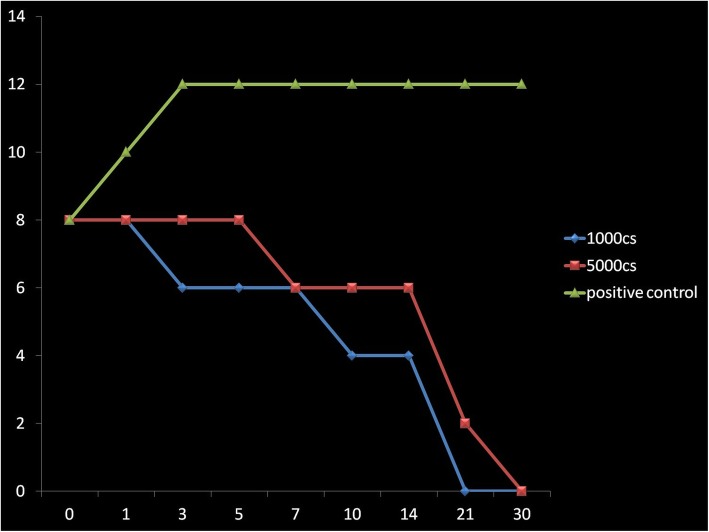
Graph showing the growth pattern of *Pseudomonas aeruginosa*. *X*-axis is in days, *Y*-axis has CFU (1 × 10^10^)

**Fig. 5:**
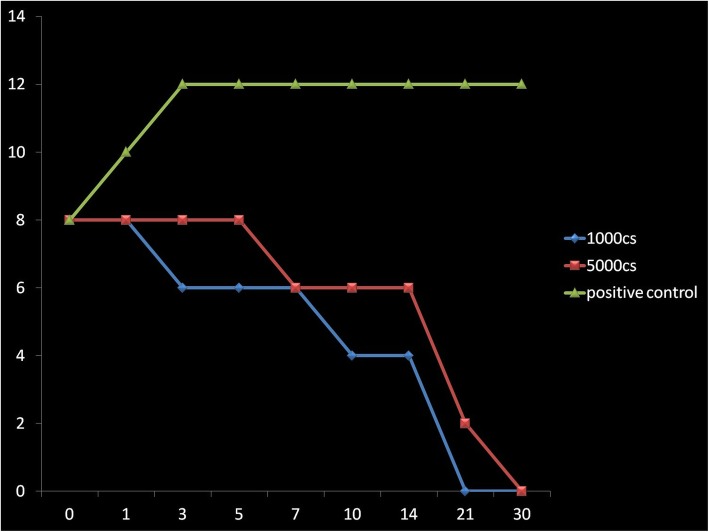
Graph showing the growth pattern of *Staphylococcus aureus*. *X*-axis is in days, *Y*-axis has CFU (1 × 10^10^)

**Fig. 6:**
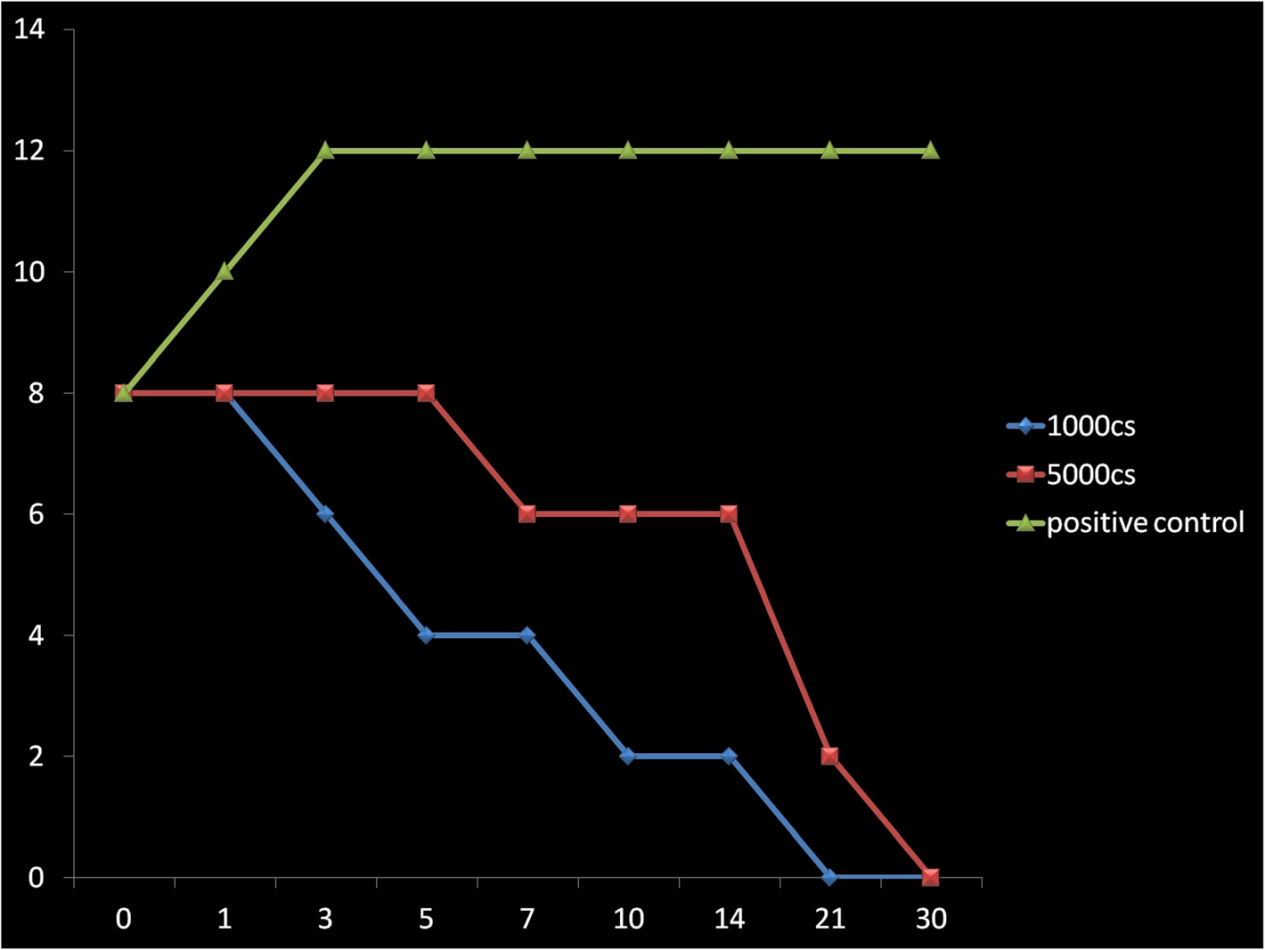
Graph showing the growth pattern of *Staphylococcus epidermidis*. *X*-axis is in days, *Y*-axis has CFU (1 × 10^10^)

In the BHI/SB enrichment medium, bacteria and fungi were observed to survive for the whole 30-day period of testing (Figs. 1, 2, 3, 4, 5, and 6) with increase in their initial load. Comparatively, Fungi did demonstrate a fungistatic action with no increase in the number of CFUs with time. The negative control SO without microorganism inoculation did not show any growth throughout the study period. Among the two types of SO used, the 1000 cSt SO showed a better inhibition of the microbial activity as compared to the 5000 cSt SO. Graphs 1, 2, and 3 show the various quanta of organisms found at different time points along the study.

## Discussion

The current in vitro study demonstrated that exposure to SO inhibited the growth of all common organisms usually implicated in endophthalmitis which possess a bactericidal action and a fungistatic action. For 1000 cSt SO, complete bactericidal action was noted by day 21 of exposure, and for the 5000 cSt SO, it was noted by day 30. Both the oils show a similar fungistatic action.

Ozdamar et al. in 1999 [4] reported nearly similar antimicrobial activity of 133 cSt silicone oil on the 7th to the 21st day. Chrapek et al. [10] reported the antimicrobial activity of higher viscosity silicone oil as early as 2 days for bacteria and 2 weeks for fungi. Neither study have used the silicone oil commonly used in clinical practice—1000 cSt and 5000 cSt. While Ornek et al. [11] observed that the antimicrobial activity increased with increase in silicone oil viscosity, Adams et al. [12] failed to demonstrate any antimicrobial activity in any of the tested silicone oil.

The endophthalmitis microbial spectrum in India is much different from that mentioned in the western literature. Along with the relatively high number of gram-negative isolates, the incidence of antibiotic resistance is also high in this part of the world [9, 13–15]. In such a clinical situation, any additional intervention which can help mitigate microbial proliferation would have a bearing on the final outcome. An important strength of the current study as compared to the previous ones is that the strains of *Klebsiella* and *Pseudomonas* that were tested were multi-drug resistant and that of *Staphylococcus* were methicillin resistant. The fact that even multi-drug resistant organisms were inhibited by SO points towards a definite role of SO in the management of these infections. Fungal growth though not inhibited completely was unequivocally halted by the presence of SO. This can also be hypothesized to be helpful in controlling fungal endophthalmitis as the organism load proliferation is avoided which can help the antifungal antibiotics injected in the vitreous cavity to have a better efficacy.

The results in our study indicate that strains of bacteria responsible for infective endophthalmitis fail to survive in silicone oil for longer than 30 days, but fungi can persist. A different point of view can be that the in vitro conditions do not reflect adequately the in vivo context of silicone oil, which is in direct contact with a variable amount of vitreous cavity fluid that is highly oxygenated. Thus, though this study does not allude to a direct bactericidal effect of silicone oil all by itself, it does point to a definite additive role of silicone oil to the standard of care which is intravitreal antibiotic injections.

## Conclusion

In conclusion, our study demonstrates a beneficial role of SO in inhibition of microbial proliferation in the vitreous cavity. A comparative prospective clinical study can help further vindicate these results. Meanwhile, we propose that in cases of infectious endophthalmitis where pars plana vitrectomy is contemplated, especially where the clinical features or previous microbiologic evaluation suggests a multi-drug resistant organism or a fungal etiology, SO tamponade may be considered at the end of vitrectomy.

## Data Availability

Please contact author for data requests.
